# Inflammatory microRNAs in cardiovascular pathology: another brick in the wall

**DOI:** 10.3389/fimmu.2023.1196104

**Published:** 2023-05-18

**Authors:** Laura Zapata-Martínez, Sonia Águila, Ascensión M. de los Reyes-García, Salvador Carrillo-Tornel, María L. Lozano, Rocío González-Conejero, Constantino Martínez

**Affiliations:** Department of Hematology, Morales Meseguer University Hospital, Centro Regional de Hemodonación, Universidad de Murcia, IMIB Pascual Parrilla, Murcia, Spain

**Keywords:** microRNAs, cardiovascular diseases, inflammation, neutrophil extracellular traps (NET), thromboinflammation

## Abstract

The regulatory role of microRNAs (miRNAs) is mainly mediated by their effect on protein expression and is recognized in a multitude of pathophysiological processes. In recent decades, accumulating evidence has interest in these factors as modulatory elements of cardiovascular pathophysiology. Furthermore, additional biological processes have been identified as new components of cardiovascular disease etiology. In particular, inflammation is now considered an important cardiovascular risk factor. Thus, in the present review, we will focus on the role of a subset of miRNAs called inflamma-miRs that may regulate inflammatory status in the development of cardiovascular pathology. According to published data, the most representative candidates that play functional roles in thromboinflammation are miR-21, miR-33, miR-34a, miR-146a, miR-155, and miR-223. We will describe the functions of these miRNAs in several cardiovascular pathologies in depth, with specific emphasis on the molecular mechanisms related to atherogenesis. We will also discuss the latest findings on the role of miRNAs as regulators of neutrophil extracellular traps and their impact on cardiovascular diseases. Overall, the data suggest that the use of miRNAs as therapeutic tools or biomarkers may improve the diagnosis or prognosis of adverse cardiovascular events in inflammatory diseases. Thus, targeting or increasing the levels of adequate inflamma-miRs at different stages of disease could help mitigate or avoid the development of cardiovascular morbidities.

## 1 Introduction

In the early 2000s, inflammation was considered an important pathophysiologic element in the initiation and progression of atherosclerosis (1). During this time, an extensive amount of evidence established that atherosclerosis was also an inflammatory process. Because atherogenesis is a cumulative process that occurs over decades, it is not easy to establish timelines of atherogenesis and inflammation. In this context, inflammation fuels the thrombotic complications linked to atherosclerosis. This new connection of separate processes (atherosclerosis, thrombosis, and inflammation) was given pathophysiological meaning in 2013, when the term immunothrombosis grouped these processes under the same umbrella (2). Immunothrombosis, which is the pathophysiological activation of coagulation in response to infectious (bacteria, virus) or sterile (atherosclerosis, cholesterol crystals, plaque rupture, blood flow stasis) stimuli, highlights the importance of neutrophils and platelets in these processes. Thus, innate immunity is functionally incorporated into the feedback established by atherosclerosis and inflammation to explain the pathophysiology of thrombotic complications in all related cardiovascular diseases. Moreover, when immunothrombosis exceeds a threshold, thromboinflammation occurs (3).

As the participation of inflammation has been confirmed, interest in inflammation as a therapeutic target in cardiovascular thrombotic complications has been increasing. This consideration has been encouraged because therapies aimed at controlling classic risk factors (hypertension, hypercholesterolemia, etc.) have not been sufficiently effective. In fact, several clinical trials are currently testing targets of inflammatory processes in cardiovascular disease (4). However, these therapies have potential harmful effects because immunosuppressive consequences such as increased fatal infections, sepsis or pneumonia have already been reported (3). Cardiovascular medicine requires the identification of new pathways and targets to improve disease prevention and follow-up.

Within this novel perspective, microRNAs (miRNAs) have begun to be examined as new modulators of thromboinflammatory processes. Since their discovery in 1993, miRNAs have been recognized as regulators of a multitude of biologic and pathologic processes. miRNAs are short double-stranded RNA molecules (~22 nucleotides) that posttranscriptionally regulate the expression of genes (5). The crucial effects that miRNAs have on inflammatory responses were demonstrated by genetic disruption of their biogenesis in macrophages (6). The authors demonstrated that several miRNAs coordinated the transcription of cytokines when these cells were activated by Toll-like receptor (TLR) ligands. Since their pathophysiological role is evident and they are key elements in cardiovascular diseases, we intend to review the role of the most relevant miRNAs that regulate inflammation (termed inflamma-miRs (7)) in the development of cardiovascular pathology, which is a thromboinflammatory disease.

## 2 Biogenesis and function of miRNAs

Excellent reviews have previously described miRNA biogenesis in detail (5, 8). Very briefly, miRNA maturation starts in the nucleus, where RNA polymerase II transcribes the miRNA gene to form pri-miRNAs. An additional processing step by a catalytic complex composed of Drosha Ribonuclease III (DROSHA) and DGCR8 Microprocessor Complex Subunit (DGCR8) yields pre-miRNA. At this point, pre-miRNA, which is a 3´ overhang hairpin-like molecule of ~60 nucleotides, is exported to the cytoplasm by a RanGTP-dependent exportin (XPO5). Pre-miRNAs are processed by DICER to form ~22 nucleotide mature double strand miRNAs. Mature miRNA is then unwound, and the thermodynamically more stable strand (named -3p or -5p) enters a macromolecular ribonucleoproteic RNA-induced silencing complex RISC to recognize a small sequence typically in the 3’-UTR of the mRNA target by base pairing. Thus, miRNA/mRNA binding will result in mRNA silencing by the inhibition of translation or by mRNA decay. However, additional parameters related to secondary structure or sequence can account for miRNA/mRNA interactions. Non-canonical seed regions in the 3’- or 5’-UTR or in the coding region of the mRNA can be functional, and one mRNA can be targeted by several miRNAs, which adds complexity to the algorithms that predict binding sites (9). These predictions also take into account the energy of the interaction, yielding thousands of putative targets per miRNA. To date, 2654 mature miRNAs (MiRBase, Release 22.1: October 2018) have been described in humans that may regulate a large part of the human transcriptome (up to 70%). Thus, the discovery of miRNAs revolutionized knowledge about the posttranscriptional regulation of eukaryotic genes. It was soon determined that the functional consequences of this new regulation are extensive and related to a multitude of physiological and pathological processes in almost all living organisms.

In this review, we will describe the dual roles of miRNAs in activating or repressing genes in inflammatory pathways and the consequences in cardiovascular diseases, as well as their roles in ensuring a return to homeostasis.

## 3 miRNAs as regulators of inflammation

The transcription of miRNAs in different pathophysiological processes is regulated by transcription factors that are cell- and stimulus-specific (10). When an acute infection occurs, inflammation quickly coordinates protection and a return to homeostasis. This balance is achieved by dynamically regulating pro- and anti-inflammatory pathways and mediators. The role of miRNAs in inflammation was reported in *Dicer*-knockout macrophages in which the depletion of miRNA biogenesis reduced the release of inflammatory cytokines after TLR activation (6). A comprehensive overview of the involvement of miRNAs in the regulation of innate immune and inflammatory responses can be found in Nejad et al. (11).

There are several reasons suggesting that miRNAs are ideal immunomodulators of inflammation. miRNAs moderately regulate translation, rarely more than 2-fold (12), which is useful in fine-tuning responses at all times. Given their small seed sequence size, the net effect on one pathway is probably the result of the cooperation of several miRNAs targeting the pathway at multiple levels. In addition, a given mRNA may be targeted by several miRNAs, thus adding to the final functional effect (11). However, the consequences of such mild regulation on related inflammatory pathways have been described. To add another degree of complexity, miRNAs might also control the switch from a strong early proinflammatory response to sustained subclinical inflammation or even to the resolution of inflammation. Finally, it has been suggested that miRNAs are differentially expressed in response to shear rate, which would modify the phenotype of cells within vessels or at distance through extracellular transport (13).

## 4 miRNAs in inflammatory cardiovascular diseases

Over the last decade, a number of reports have demonstrated functional links between miRNAs and cardiac function. Substantiating this link, Chen et al. demonstrated that cardiac knockout of *Dicer* causes dilated cardiomyopathy, heart failure, and postnatal lethality (14). Additionally, conditional *Dicer* deletion in the postnatal myocardium resulted in spontaneous cardiac remodeling, hypertrophy, and fibrosis (14, 15). Other studies also showed the role of miRNAs with a similar strategy. Hartmann et al. constructed a model of Dicer deficient endothelial cells and observed an increased atherosclerosis in part mediated by the regulation of KLF4 by miR-103 (16). Vascular smooth muscle cells (VSMC) also played a relevant role in cardiovascular diseases. The importance of miRNAs in these cells, was also demonstrated in a study employing a conditional mouse model of Dicer deficiency in VSMC (17). The authors showed that *Dicer* deletion in VSMCs plays an essential role in vascular repair since the lack of anti-proliferative miRNAs such as miR-27a-3p that help inhibiting vessel stenosis, fail to protect Dicer^-/-^ mice against neointima formation processes induced by wire-induced injury to carotid arteries. Accordingly, these works suggest that miRNAs play an essential role not only in cardiac function physiology but also in cardiovascular diseases (CVD).

Here, we selected six inflamma-miRs that, according to published data, may be considered the most representative candidates to play a functional role in thromboinflammation in CVD: miR-21, miR-33, miR-34a, miR-146a, miR-155, and miR-223.

### 4.1 miR-21 (the essential pawn)

miR-21 is an important mediator of many inflammatory pathways that are activated by TLR signaling (18). First established as an onco-miR, which is a miRNA with a functional role in cancer, miR-21 has been further described as a crucial element in the control of inflammatory responses. Thus, Sheedy et al. reported that by regulating programmed cell death protein 4 *(PDCD4)* expression, miR-21 modulated the response to LPS by inhibiting *NF-κB* activation and promoting *IL10* expression (19). Additionally, miR-21 has been shown to be induced by the anti-inflammatory factor resolvin-D1 (20). In contrast, miR-21 overexpression is related to autoimmune diseases, suggesting that this miRNA establishes an anti-inflammatory and immunosuppressive milieu (21). In a colitis model, miR-21-knockout mice have lower proinflammatory cytokines and longer survival times than wild-type mice (22). These dual effects allow miR-21 to control a potentially lethal response called “LPS tolerance”. Thus, miR-21 represents the paradigm of the context-dependent effects of a single miRNA, which regulates the inflammatory switch.

The involvement of miR-21 in CVD is well established, particularly when cardiac dysfunction is purely associated with inflammatory processes, as is the case for sepsis. Up to 50% of patients with sepsis develop heart disease, and in those with severe sepsis and septic shock, septic cardiomyopathy is a frequent complication that compromises patient survival (23). Wang et al. described the participation of miR-21 in this pathology in a murine model of cardiac dysfunction induced by systemic administration of LPS (24). miR-21 was increased in the myocardium of LPS-injected mice, and mimic and antagomiR strategies corroborated the association between miRNA levels and cardiac dysfunction. Furthermore, the authors suggested that Sorbin And SH3 Domain Containing 2 *(SORBS2)* is the miR-21 target that regulates septic cardiomyopathy (24).

On the other hand, a major role for miR-21 in atherogenesis by regulating several cells and signaling pathways has been described. Shear stress-induced endothelial cell (EC) inflammation has been shown to be mediated by transcriptional silencing of *PPARα* by miR-21 (25). In addition, ECs overexpressing miR-21 enhanced the Akt/PKB signaling pathway, leading to nitric oxide (NO) production, proliferation, and apoptosis in cells (26). In other cell types, Wei et al. showed that miR-21 could regulate VSMC phenotype, facilitating their migration and proliferation from the media to the intima (27). More recently, Canfran-Duque et al. demonstrated the contribution of miR-21 in macrophages to atherogenesis (28). By using a model of low density lipoprotein receptor (Ldl)r^-/-^ mice transplanted with bone marrow (BM), the authors demonstrated that mice with miR-21^-/-^ BM developed larger atherosclerotic areas. Genetic ablation of miR-21 in macrophages decreased ABCG1 expression, which contributed to foam cell formation (28).

Other studies have evaluated miR-21 expression in cardiac tissues *ex vivo*. Parahuleva et al. (29) analyzed the expression of 352 miRNAs in human advanced coronary atherosclerotic plaques and found that miR-21 was the miRNA with the highest upregulation. High miR-21 expression is related to a more advanced inflammatory state and therefore greater plaque instability. Thus, increased expression of miR-21 in carotid plaques from the endarterectomy specimens of patients with symptomatic versus asymptomatic plaques was reported by Markus et al. (30).

Additional studies have assessed the therapeutic effects of anti-miR-21 on in-stent restenosis (ISR) models. Wang et al. used anti-miR-21-coated stents in a myointimal hyperplasia/ISR mouse model and observed an efficient reduction in ISR compared with bare-metal stents while avoiding systemic anti-miR-21 off-target effects (31). In humans, plasma levels of miR-21 can predict vascular restenosis in lower extremity arterial occlusive disease 6 months after interventional therapy (32).

In summary, miR-21 has an essential role on the switch of the inflammatory response. In cardiac tissues, its expression positively correlates with atherosclerosis, plaque instability, and restenosis.

### 4.2 miR-34a (the moonlighter)

Similar to miR-21, miR-34a was first described as an onco-miR (33). miR-34a regulates sirtuin 1 (SIRT1) expression, which affects the cell cycle and apoptosis by increasing acetylated p53 while inducing the expression of p21 and p53 upregulated modulator of apoptosis (PUMA). PUMA and p21 are targets of p53 and regulate apoptosis and the cell cycle, respectively. By closing a positive feedback loop, p53 regulates the expression of miR-34a (33). Since these processes are fundamental for cellular homeostasis, miR-34a was later described as an inflamma-miR based on its role in regulating autophagy, ROS generation or fibrosis. In addition, the participation of miR-34a in these cellular processes identified it as an essential regulator of CVD (34). Several studies have reported *in vitro* and/or *ex vivo* results from cellular or animal models assessing the functional role of miR-34a in related-CVD pathways:

In angiogenesis, the effect of miR-34a seems to be cell- and context-dependent. It has been reported that this miRNA exacerbates apoptosis and inhibits angiogenesis *via* Notch1 signaling in cardiac cells within the microvasculature (35).

In a model of induced hypoxia, miR-34a was increased in cardiomyocytes, and knockdown of this miRNA upregulated zinc finger E-box binding homeobox 1 (ZEB1) and decreased apoptosis (36).

In atherosclerosis, it has been reported that miR-34a modulates ATP binding cassette subfamily A member 1 (ABCA1) expression in macrophages, which could have direct effects on cholesterol efflux and reverse cholesterol transport in these cells (37). Furthermore, these authors showed that selective ablation of miR-34a in macrophages inhibited atherosclerosis progression in an Apolipoprotein (Apo)E^-/-^ mouse model (37).

In VSMCs, *in vitro* models showed the participation of miR-34a in the function of these cells and in neointima hyperplasia. Therefore, in vascular remodeling, which is a pathology in which carotid intima-media thickening is decisive, VSMC proliferation and migration are regulated by miR-34a *via* Notch1 expression control. These authors showed that proliferation and migration were decreased with miR-34a overexpression and increased with miRNA ablation (38).

In calcific aortic valve disease (CAVD), which severely affects more than 3% of the population over 75 years of age, the expression of miR-34a, among other miRNAs, was found increased in the valve tissues of individuals with CAVD *vs.* those from patients with aortic regurgitation. Consistently, in CAVD samples, underexpression of Notch1 and overexpression of Runx2 were also found by comparing the samples in this way (39).

In cardiac fibrosis, Huang et al. showed in a mouse model that miR-34a was upregulated after myocardial infarction. In this context, cultured cardiac fibroblasts showed a direct correlation between miR-34a levels and transforming growth factor (TGF) β1 activity, suggesting that the participation of miR-34a in cardiac fibrosis involves targeting Smad4 expression (40).

Overall, miR-34 is a key element in cellular homeostasis therefore, increased miR-34 levels have been extensively related to pathologic cardiac phenotypes.

### 4.3 miR-33a/b (the double-sided lipid keeper)

*MIR33A/B* genes are highly conserved between species and are located in intronic regions of Sterol Regulatory Element Binding Transcription Factor (*SREB) F2/1*; these two transcription factors are induced when lipids are scarce and activate genes involved in cholesterol, fatty acid, and phospholipid synthesis. In humans, miR-33a is highly expressed in peripheral blood mononuclear cells, while the isoform miR-33b is most highly expressed in the liver (41). In mice, there is one isoform of miR-33, and it is necessary to take this into account when extrapolating the results obtained in mice to other models. In this review, we will refer to miR-33 generically.

Functionally, miR-33 targets the genes responsible for cholesterol transport, such as *ABCA1* and ATP binding cassette subfamily G member 1 (*ABCG1)*, as well as several transcripts encoding proteins involved in fatty acid β-oxidation, such as carnitine palmitoyltransferase 1A (CPT1A), hydroxyacyl-CoA dehydrogenase trifunctional multienzyme complex subunit beta (HADHB) and carnitine O-octanoyltransferase (CROT). Therefore, these loci are master regulators of cellular cholesterol metabolism (42). Cholesterol efflux from macrophages and hepatocytes can maintain appropriate lipid levels within the cell and is mainly mediated by *ABCA1/G1.* In brief, ABCA1/G1 interacts with apoA1 to begin HDL biogenesis. Since HDL efflux capacity is directly correlated with a lower CV risk (43), it would be expected that low expression of miR-33 and high levels of ABCA1/G1 would be atheroprotective. However, recent works have demonstrated that knocking out *Mir33* has diverse and unexpected effects depending on the cell type and the microenvironment. Price et al. found that complete miR-33 deficiency in the Ldlr^-/-^ mouse model favored obesity, hyperlipidemia, and insulin resistance without affecting plaque development (44). Specific macrophage miR-33^-/-^ in that mouse model reduced lipid deposits and inflammation, which were accompanied by a reduced plaque burden (44). Shortly after, these authors showed that conditional miR-33^-/-^ in the liver did not result in obesity in hyperlipidemic conditions; rather, the mice were resistant to hepatic fibrosis and insulin resistance (45).

In addition to atherosclerosis, miR-33 is related to cardiac fibrosis, which is a markedly inflammatory pathophysiological condition that occurs after heart injury. In this context, cardiac fibroblasts acquire a hyperproliferative phenotype and release proinflammatory and fibrotic proteins, which can result in pathological cardiac remodeling (46). Nishiga et al. described the first evidence of the effects of miR-33 and its target ABCA1 on the proliferation of cardiac fibroblasts and the adaptive response in the remodeled heart (47). Consistent with previous observations by Price et al. (45), these authors reported that in cardiac fibrosis, specific miR-33 deficiency in cardiac fibroblasts can act as a double-edged sword because decreasing cardiac fibrosis also promotes heart failure (47). These results indicate that to ensure the success of miR-33 inhibition as an antiatherosclerosis tool, cell- or tissue-specific targeting of miR-33 should be considered.

Although these data would be sufficient to justify the relevance of miR-33 in CVD, there are other data showing an additional role of miR-33 in inflammation. Several animal models have shown that the regulation of cellular cholesterol homeostasis can modulate the inflammatory response beyond atherosclerosis. Clear examples have shown that *ABCA1* expression has other effects on many organs and tissues, such as affecting amyloid burden and inflammation in neurons and glia (48) or contributing to myeloid precursors and the activation of granulocytes (49).

In CVD, crosstalk between TLR4 activation, liver X receptor and ABCA1 expression in macrophages has been shown to modulate inflammatory gene expression *via* NF-κB [an in-depth review detailing these complex interactions can be found in Westerterp et al. (50)]. Mechanistically, inefficient lipid efflux causes lipid accumulation within cellular membranes, which mobilizes TLRs to lipid rafts and enhances MyD88-dependent TLR inflammatory responses (51). It has also been shown that the ABCA1/apoA1 interaction in macrophages activates the JAK2/STAT3 pathway, which is independent of lipid flux control (52). In murine miR-33^-/-^ macrophages, Price et al. identified changes in the expression of a set of genes that may be associated with the inhibition of inflammatory signaling through the NF-κB and TLR4-mediated pathways (44).

Taken together, these data position miR-33 in the tissue-specific context in which the functional effect of a miRNA must be understood. This idea is especially relevant when looking for therapeutic options that involve miR-33 in dyslipidemia, obesity or atherosclerotic plaque progression.

### 4.4 miR-146a (the inflammatory brake)

In 2006, Taganov et al. published their seminal study, which demonstrated that the expression of miR-146a was induced by NF-κB in a negative feedback loop that downregulated the protein levels of IL-1 receptor-associated kinase 1 (IRAK1) and TNF receptor–associated factor 6 (TRAF6) (53). These authors showed that bacterial compounds induced NF-κB through a MyD88-dependent pathway; in turn, miR-146a was upregulated. Interestingly, increased levels of miR-146a fine-tune inflammatory cytokine production rather than completely override this pathway. Shortly after, this same group characterized the role of miR-146a in immune and inflammatory responses in a mouse model (54). Ablation of miR-146a in mice resulted in exaggerated inflammation in response to endotoxemic challenge. Later in life, miR-146a^-/-^ mice developed multiorgan inflammation, myeloid proliferation and cancer, leading to premature death (54). These data indicate an additional pathway to link inflammation and CVD.

Thus, a functional role for miR-146a in ischemic/reperfusion (I/R) injury has been described. Xiao et al. injected a miR-146a-mimic into a mouse model before I/R injury and preserved cardiac function, reduced the infarct zone and fibrosis and decreased the inflammatory response (55). Similarly, Su et al. reported that specific ablation of miR-146a in cardiomyocytes worsened the consequences of I/R injury, such as myocardial infarction, apoptosis and cardiac dysfunction. Mechanistically, these authors described a novel function for miR-146a, which translocates into mitochondria to regulate mitochondria-dependent apoptotic pathways in cardiomyocytes. Thus, in response to I/R stress, miR-146a mitochondrial levels decreased, leading to mitochondrial dysfunction and cardiomyocyte apoptosis (56).

The characterization of the role of miR-146a in atherosclerosis has been challenging. Several studies have described a paradoxical relationship between this miRNA and atherogenesis, which might depend on the cellular compartment on which the study be focused. Thus, Petrkova et al. reported that miR-146a expression was significantly higher in heart valves with atherosclerosis than in those without atherosclerosis (57). In the same line, Cheng et al. used a double-knockout model of fat-fed Ldlr^-/-^ & miR-146a^-/-^ mice and found a reduction in atherosclerosis compared to the single Ldlr^-/-^ model but unexpectedly, an increase in proinflammatory cytokines (58). To explain these results, the authors investigated the cell-specific contribution to atherosclerosis by separating the contribution of bone marrow (BM)-derived cells. Transplantation of BM wild-type in the Ldlr^-/-^ and double-KO models revealed that the latter had an elevated atherosclerotic plaque burden compared with the former. Consistently, ablation of miR-146a specifically in BM decreased atherogenesis, while ablation in the vasculature alone improved atherogenesis (58). Using a similar approach, our group demonstrated that Ldlr^-/-^ mice with BM-miR-146a^-/-^ did not develop accelerated atherosclerosis in comparison with mice that were transplanted with BM-WT after 20 weeks of fat feeding (59). These results suggest that the lack of miR-146a, is atheroprotective in BM but atherogenic in endothelium in these mouse models. Even though there is a specific cellular functional effect for miR-146a (as for other miRNAs), these studies have been carried out in animal models in which cholesterol undoubtedly plays a crucial role (interestingly, *Abca1* is a target of miR-146a in *in silico* predictions using www.targetscan.org). In addition, miR-146a-KO mice might not be the best model to study atherosclerosis with a moderately prolonged time because the myeloproliferative phenotype and BM exhaustion are unmasked and could be confounding factors when drawing definitive conclusions.

Additional bibliographic data can add several levels of complexity. First, it has been shown that levels of miR-146a and its role in atherogenesis could be modulated by the nature of blood flow. Chen et al. examined injured rat carotid arteries and showed that miR-146a (among others) was highly expressed in neointimal lesions under physiological levels of flow but not under stasis conditions (60). Second, the transcription of miR-146a can be regulated by several functional polymorphisms (miR-SNPs), some of which are related to susceptibility to several diseases. Lofgren et al. confirmed that rs2431697, a functional miR-SNP located in the intergenic region between the pituitary tumor transforming gene 1 (*PTTG1)* and *MIR146A* genes that reduces the expression of miR-146a by 50%, was significantly associated with systemic lupus erythematosus (SLE) in a population of 1324 patients and 1453 controls (61). The relationship between functional miR-SNPs, particularly rs2431697, and CVD are outside the focus of this review; more details can be found in a recent review from our group (62).

In summary, miR-146a is a natural brake on the NF-κB pathway. However, in deficient mice models, the inherent myeloproliferative phenotype in BM implies that its expression has a tissue-dependent effect. In humans, its partial deficiency due to rs2431697 has been linked to worse CVD outcomes.

### 4.5 miR-155 (the cooperator)

miR-155 is a classic inflamma-miR that plays important roles in innate and adaptive immunity. Its coding sequence is located within the host gene *MIRHG155* (the B-cell Integration Cluster -BIC- gene), whose expression is regulated by transcription factors such as NF-kB, SMAD4, ISRE, IRF, LXRα and AP-1 (63–65). Some dietary compounds (polyunsaturated fatty acids, arachidonic acid) can inhibit miR-155 expression (66). Furthermore, the transcription of miR-155 fluctuates in response to several inflammatory signals: TNFα, IL1β, pathogens associated molecular patterns (PAMPs), Damage associated molecular patterns (DAMPs), TLR ligands, alarmins or hypoxia (67).

Despite this clear anti-inflammatory role, *in vitro* studies have yielded contradictory results about whether miR-155 mediates pro- or anti-inflammatory functions in monocytes/macrophages. In fact, although it has been accepted that miR-155 overexpression favors a shift in macrophages to the M1 phenotype, while its downregulation leads to the M2 phenotype, the correlation between miR-155 expression and inflammation is not direct; rather, it seems to be dependent on the tissue, animal model, state of development, and disease (66). Thus, in an exhaustive work, Hsin et al. showed that miR-155 differentially regulated identical 3′-UTR isoforms of genes in key immune cells (macrophages, dendritic cells, and T and B lymphocytes), suggesting that this miRNA exerts its effects depending on the cellular context (68).

Adding complexity to the interpretation of its functional effect, miR-155 is a good example of a miRNA with a gene regulation network that cooperates with each other to achieve more precise control. Thus, miR-155 and miR-146a are associated in a regulatory network that controls NF-κB activity in mouse macrophages. miR-155 and miR-146a regulate the intensity and duration of the inflammatory response in a two-stage process with a negative feedback loop (69). To develop a complete picture of how complicated the functional context of miR-155 can be, it has been shown that miR-155 deletion in murine macrophages significantly disturbs the circadian effects on cytokine responses to LPS (70). Mechanistically, these authors demonstrated that the molecular clock acts as a miR-155 switch that represses Basic Helix-Loop-Helix ARNT Like 1 (BMAL1) so that the innate immune response in myeloid cells adjusts to the variability of the circadian rhythm (70).

In the context of CVD, abundant evidence demonstrates the effects of miR-155 on cellular and animal models. For example, Park et al. showed that miR-155 negatively regulated the contractile capacity of VSMCs and vasorelaxation (71). Wu et al. showed that the inflammatory response of macrophages to ox-LDL was mediated by the effect of TIR-domain-containing adapter-inducing interferon-β (TRIF) on BIC/miR-155 through the ERK signaling pathway (72). More recently, Peng et al. described a role of the miR-155/NF-κB axis in activating the NLR family pyrin domain containing 3 (NLRP3) inflammasome *via* the ERK signaling pathway in a fat-fed ApoE^-/-^ mouse model (73). Regarding most classical mechanisms of atherosclerosis, miR-155 has been related to cholesterol efflux mediated by ABCA1/ABCG1, which is a key process in macrophage foam cell formation. Thus, in an ApoE^−/−^ mouse model, Wang et al. demonstrated that miR-155 regulated Abca1/Abcg1-dependent cholesterol efflux in macrophages, promoting their change to the M2 phenotype (64). Mechanistically, these authors showed that the overexpression of Ctrp12 (a conserved paralog of adiponectin) decreased miR-155 levels, which increased Abca1/Abcg1 activity to inhibit lipid accumulation and the inflammatory response in macrophages (64). In addition, Zheng et al. (among others) reported an interesting association between miR-155 and T-cell-based immunity and atherogenesis (74). Thus, the results from these groups show that miR-155 promotes T-cell proliferation and regulates cytokine production, resulting in an immune tolerance mechanism that accounts for atherosclerosis development (70, 74, 75).

In human samples, data on miR-155 expression in atherosclerotic lesions are scarce and suggest a proinflammatory role of miR-155 in pathological tissues. Li et al. analyzed miR-155 expression in pairs of samples (atherosclerotic lesions and healthy areas) from 17 patients who underwent angiography. Their results showed significantly higher miR-155 levels in atherosclerotic lesions than in the healthy veins of the same subjects (63).

Finally, in addition to atherogenesis, the expression of miR-155 has been associated with the pathophysiological processes that take place after the occurrence of acute myocardial infarction (AMI). After AMI, miR-155 is dynamically increased in murine hearts, and so a protective role of transient miR-155 silencing in cardiac dysfunction and remodeling shortly after the event has been reported. Using a mouse model of AMI, Guo et al. showed that the downregulation of miR-155 decreased apoptosis in cardiomyocytes (76). In addition, Hu et al. showed that miR-155 silencing in an AMI mouse model resulted in less inflammation in macrophages and attenuated the activity of the Suppressor of Cytokine Signaling 1 (Socs1)/Nf-κb pathway, which decreased endoplasmic reticulum stress-induced cardiomyocyte apoptosis (77).

Overall, miR-155 *per se* has a clear anti-inflammatory role. However, it is also an illustrative example of the coordinated effect exerted by miRNAs, balancing their effects to achieve a net action that conveniently controls the inflammatory response. Therefore, this miRNA teaches us how biased the interpretation of its effect on CVD can be if we do not take into account the cell and tissue specificity of miRNAs.

### 4.6 miR-223 (the traveler)

The *MIR223* sequence is located on the X chromosome and is highly conserved among species. The expression of miR-223 has been described in myeloid cells, hepatocytes, and cardiomyocytes. miR-223 dysregulation was first associated with cancer and was more recently extended to inflammation, immune response, and cardiovascular diseases. miR-223 has a crucial role in myeloid cell differentiation through several transcription factors that regulate its expression, such as PU.1, CCAAT-enhancer-binding proteins (C/EBP)-α and -β and nuclear factor I-A (NFI-A) (78). However, similar to other miRNAs, this regulation is context dependent, and miR-223 is downregulated in the early phases of granulocyte-monocyte progenitor differentiation and upregulated when these cells enter the differentiation phase. Moreover, miR-223 participates in megakaryocyte differentiation, osteoclastogenesis, and the differentiation of human embryonic stem cells (78). In an inflammatory context, miR-233 targets a conserved sequence within the NLRP3 3´UTR to prevents IL-1β production by inflammasomes in response to cell activation through TLRs and during myeloid differentiation (79).

In recent years, miR-223 has been linked to several pathophysiological processes related to CVD, and there has been increasing interest in its use as a diagnostic and/or therapeutic tool. Its abundance in platelets and the secretory function of these cells supported the growing interest in the role of platelet miR-223 as an extracellular miRNA that exerts biological effects on recipient cells to regulate their activity (80). In a similar context, an interesting mechanism consisting of the formation of HDL/miRNA complexes (the most abundant being HDL/miR-223 in familial hypercholesterolemia) has been described in the control of cholesterol homeostasis. Cuesta-Torres et al. showed that miR-223 was present in HDL and originated from myeloid cells through a process in which plasma HDL activates miR-223 transcription by SR-BI-induced lipid flux in a feedback loop and the transfer of miR-223 to the lipoprotein (81).

Thus, miR-223 synthesized by monocytes, macrophages and/or platelets is available in plasma to act as an endocrine factor (82) and for cell−cell intercommunication between these cells and endothelial cells or VSMCs, where it may exert different effects (83). In these host cells, miR-223 decreases lipid deposition in the arterial wall, promotes endothelial activation, and induces vascular remodeling. At the molecular level, plasma miR-223 targets Intercellular Adhesion Molecule 1 (ICAM1) and regulates the NF-κB and Mitogen-activated protein kinase (MAPK) signaling pathways in endothelial cells (84). Similarly, exogenous miR-223 exerts effects on VSMCs through the PI3K/AKT or TLR4/NF-κB signaling pathways, decreasing vascular neointimal formation and atherosclerosis (82).

Recently, Nguyen et al. confirmed the protective role of miR-223 in atherosclerosis. These authors showed that mice transplanted with BM-miR-223^-/-^ had increased plaque sizes, lipid levels and IL-1β plasma levels. Analysis of the global macrophage RNA translation profile in BM-miR-223^-/-^ and WT mice showed the upregulation of mRNAs containing miR-223 binding sites that belong to inflammatory signaling and lipid metabolism pathways, confirming that miR-223 controls their translation (85). Similarly, You et al. measured the expression levels of miR-223 in atherosclerotic and healthy carotids in human samples and revealed that increased miR-223 levels were associated with stable lesions and reduced levels of atherosclerotic markers (86).

In AMI, the role of miR-223 is controversial since some studies show that it decreases I/R-induced inflammation and necroptosis in cardiomyocytes, while other studies show an association between miR-223 levels and fibrosis and arrhythmia induced after AMI. In a mouse model of I/R injury, Qin et al. reported higher inflammation and necroptosis in pre-miR-223^-/-^ hearts than in WT hearts through activation of the IKKα pathway and NLRP3 (87). On the other hand, Tang et al. showed that the absence of miR-223 decreased processes associated with hypoxia-induced oxidative stress in a cardiomyocyte model of apoptosis, caspase-3 activation or ROS generation (88). Additionally, it has been shown that miR-223 might induce myocardial fibrosis and arrhythmia, which are usually established after AMI (83).

Platelet miR-223, which is also relevant to platelets themselves, deserves special mention for several reasons: (i) it is the most abundant miRNA in platelets; (ii) it has been identified as a regulator of *P2Y12* mRNA in human platelets and may regulate platelet function (89); and (iii) it is a marker of the degree of P2Y_12_ inhibition in patients (83). P2Y_12_ is a seven-transmembrane domain receptor coupled to Gi2 protein, and it is the main ADP receptor in platelets, whereby it mediates platelet function (activation, aggregation, secretion, and thrombus stability). miR-223 may control platelet reactivity and function through the regulation of P2Y_12_. Kaudewitz et al. demonstrated this point by analyzing several markers of platelet activation in an ACS patient cohort 30 days after an acute event. These authors found a positive correlation between intraplatelet miR-223 levels, platelet function tests and several plasma markers of platelet activation (90). A more recent meta-analysis corroborated these data (91).

Regarding antiplatelet therapy, the importance of miR-223 in CVD is well known, and it not only plays a significant role in prevention but also markedly impacts the prognosis of patients. Thus, pharmacological schemes are varied, including aspirin and clopidogrel, aspirin and other P2Y_12_ antagonists, or monotherapy, and their choice will depend on the pathology and the global state of the patient. Responsiveness to the therapy (defined as conservation of the platelet response or not) has been extensively related to adverse outcomes of CVD (83). Several groups have reported the utility of miR-223 plasma levels as a marker of the efficacy of antiplatelet treatment and of the suitability of patients as candidates for receiving clopidogrel as the best prophylactic/therapeutic option. However, miR-223 levels in plasma have also been shown to be influenced by the degree of P2Y_12_ expression (89), and so the net relationship between miR-223 levels and platelet reactivity might be influenced by the pathology, the type of antiplatelet drug or even the time of administration of these drugs.

In conclusion, intraplatelet and circulating miR-223 levels are useful biomarkers of the response to antiplatelet drugs (83, 92, 93). Since miR-223 levels could determine the efficacy of antiplatelet treatments and the prognosis of patients, further studies in these areas are needed.

## 5 Inflammatory miRNAs as regulators of neutrophil extracellular trap formation

Another newly characterized role of miRNAs related to inflammation and CVD is the regulation of neutrophil extracellular trap (NET) production. Neutrophils are essential elements in the innate immune system, and they represent the major leukocyte population in humans and 10% in mice. Neutrophils are the first leukocytes that are recruited at the site of inflammation after tissue damage or an infection (94). Almost two decades ago, Brinkmann et al. described a new mechanism (NETosis) in which neutrophils eliminated bacteria by liberating their nuclear content into the extracellular space (95). NETs are composed of chromatin associated with a set of proteins, such as histones and cytoplasmic and granular antimicrobial proteins (myeloperoxidase, elastase, cathepsins, etc.), that are able to permeabilize cells (96). Thus, the main role of NETs is to trap and eliminate pathogens and avoid spreading. However, NETs can promote severe tissue damage and are involved in the pathophysiology of several diseases, such as cancer (97), thrombosis (98), and atherosclerosis (99). The mechanisms of NETosis are still under investigation, and several new elements involved in this process have yet to be discovered. miRNAs are one of the latest factors to be associated with the regulation of NET formation.

To date, ten miRNAs (miR-15b-5p (100), miR-142-3p (101), miR-144 (102), miR-146a-5p (103), miR-155 (104), mir-223 (105), miR-378a-3p (100), miR-505 (106), miR-3146 (107), and miR-4512 (108)) have been shown to regulate NETosis in mammals (109). Three of them are considered inflamma-miRs (miR-146a-5p, miR-155, and miR-223) in the present review and will be briefly described. The remaining miRNAs have not been associated with cardiovascular disease or inflammatory processes (miR-15b-5p, miR-505, miR-3146, and miR-4512), or few studies have been published [miR-142-3p (31), miR-144 (32), and miR-378a-3p (33)].

### 5.1 miR-146a

The first miRNA that was shown to regulate NET formation was miR-146a (103). Activation of neutrophils in mice deficient for miR-146a (miR-146a^-/-^) revealed a higher increase in cell free DNA (cfDNA) and citrullinated Histone H3 (citH3)-positive cells/total cell ratio than their WT littermates after stimulation with phorbol-12-myristate-13-acetate (PMA). These first results confirmed that miR-146a deficiency was involved in NET formation and modified the intrinsic capacity of neutrophils during the NETotic process. Recently, these data have been confirmed (110) *in vitro*. *In vivo* studies in mice stressed with a high-fat diet (ApoE^-/-^ x miR-146^-/-^) or induced with endotoxemia by lipopolysaccharide (LPS) (miR-146^-/-^) exhibited increased NETosis in comparison with control littermates (59, 111). We then studied the phenotype of miR-146a^-/-^ neutrophils and found an aging-like phenotype characterized as CD62L^low^ CD11b^high^ C-X-C Motif Chemokine Receptor 1 (Cxcr4)^high^ and the overexpression of Tlr4. Additionally, miR-146a^-/-^ neutrophils exhibited reduced expression of Cxcr1, which has been associated with a proinflammatory phenotype (111). We also observed increased formation of reactive oxygen species (ROS) in miR-146a^-/-^ neutrophils relative to WT neutrophils under basal conditions. These results may partially explain why miR-146a deficiency increases NET formation, since ROS have been implicated in NETosis (112), and aging-like neutrophils are more prone to form NETs (113). Overall, these results suggest that miR-146a deficiency primes neutrophils to form NETs. Whether miR-146a overexpression inhibits NET formation and may be a useful therapeutic tool to control this process in several pathologies is unclear, and it is unknown if these results are transferable to humans. The latter question may have the beginning of an answer. Our group has shown that in inflammatory diseases such as atrial fibrillation or sepsis, rs2431697, a miR-SNP located 16 kb upstream of pre-miR-146a and whose minor T allele reduces miR-146a levels by 50% (61), increases the risk of developing adverse cardiovascular events (111, 114). In the first *ex vivo* experiment in healthy controls, we observed that neutrophils from rs2431697 TT homozygous subjects had increased cfDNA levels and citH3-positive cell/total cell ratios than those from CC subjects after PMA activation, replicating the results that were previously obtained in miR-146a^-/-^ mice. Additionally, in a cohort of septic patients, we found that among patients with higher NETosis markers (DNA-citH3>4^th^ quartile), carriers of the T allele were 3-fold more frequent than CC patients. Recently, we investigated plasma levels of cfDNA and DNA-citH3 in a cohort of patients <45 years old with ACS and found that T allele carriers had significantly higher levels than C homozygous patients, but no significant association was observed between the rs2431697 genotype and any of the two markers in healthy controls (115).

Overall, these results indicate that a reduction in miR-146a in humans induces an increase in NETosis under certain pathological conditions.

### 5.2 miR-155

Two studies by the same group showed that miR-155 regulates the formation of NETs. First, Hawez et al. showed that miR-155 could affect NET formation by inhibiting the expression of peptidylarginine deiminase 4 (PAD4), a protein involved in NETosis (98, 104). The authors demonstrated that the interaction of miR-155 with the *PAD4* 3’UTR increased the levels of *PAD4* mRNA and PAD4 rather than downregulating them, as occurs for the majority of targets. The authors explained this singularity to the fact that miR-155 binds to an AU-rich element (ARE) located in *PAD4* with 6´-mer perfect binding. As shown by other authors, these AREs are critical in promoting translation of miRNA (104). Indeed, their data showed that blocking miR-155 binding site in *PAD4* 3´UTR reduced the expression of PAD4 after transfecting with a miR-155 mimic (104). Additionally, RNA immunoprecipitation assay demonstrated a direct interaction between miR-155 and *PAD4* mRNA. Thus, this interaction of miR-155 with *PAD4* mRNA led to an increase in NETosis when primary human neutrophils were transfected with a miR-155 mimic.

The latter experiments were performed *in vitro*, and the relevance of these data was recently confirmed *in vivo* using miR-155-deficient mice in a model of cecal ligation and puncture (CLP)-induced sepsis (116). The authors showed that neutrophils from miR-155^-/-^ mice intrinsically produced fewer NETs than those from WT mice after *ex vivo* activation with Cxcl2. Septic mice that were deficient in miR-155 also had lower levels of NETs in plasma, probably due to lower levels of Pad4 in circulating neutrophils, which protected them from sepsis comorbidities such as lung injury (116).

Similar to miR-146a, it would be of interest to test the potential use of anti-miR-155 as a therapeutic strategy against NETosis and evaluate the impact of rs767649, which is a miR-SNP that increases the transcriptional activity of the miR-155 gene (117), on thrombo-inflammation.

### 5.3 miR-223

There have been only two studies thus far documenting an association between miR-223 and NETosis. Liao et al. evaluated the mechanisms involved in the higher NETosis observed in patients with adult-onset Still’s disease (AOSD), a systemic autoinflammatory disease that is accompanied by neutrophilia (105). Their results concluded that miR-223 could directly influence the formation of NETs by suppressing IL-18-induced ROS production through the inhibition of calcium influx. In addition, miR-223 could indirectly reduce NETosis by suppressing macrophage IL-18 production. Thus, the authors showed that neutrophil extracellular vesicles overloaded with miR-223 were taken up by macrophages and reduced the expression of IL-18 by targeting NLRP3 (105). Ye et al. demonstrated that neutrophils from miR-223^-/-^ mice were intrinsically more prone to form NETs than WT neutrophils in response to LPS (118). The authors evaluated NETosis in a model of acute liver injury and showed that miR-223 deficiency was associated with higher mortality, higher liver neutrophil infiltration, and enhanced NET formation compared to WT. The mechanism of miR-223 in NETosis is not fully explained but seems to be part of an axis formed by this miRNA and neutrophil elastase (118).

## 6 Perspectives and conclusions

**Figure 1:**
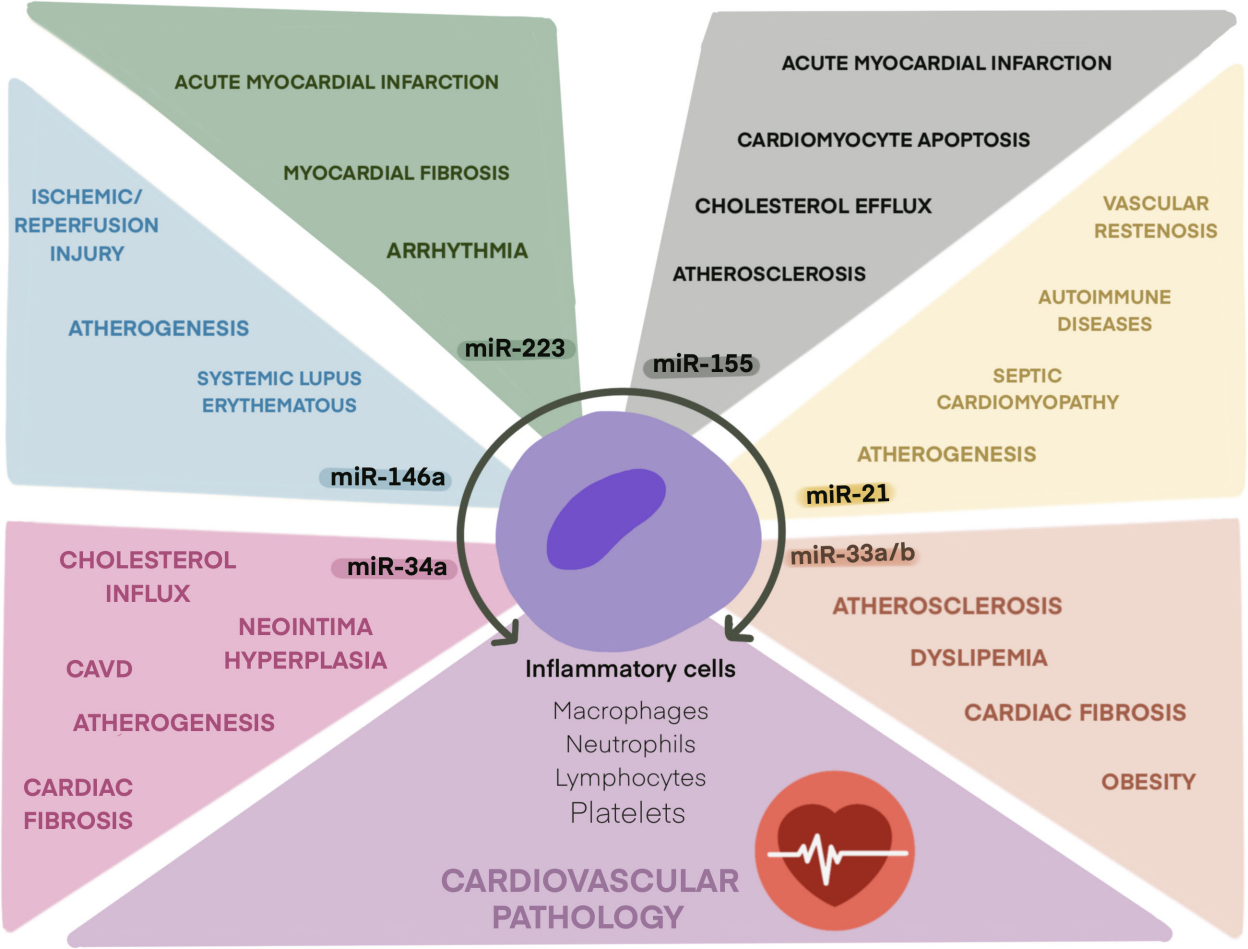
Role of main inflamma-miRs in cardiovascular pathologies. Schematic illustration of inflamma-miRs miR-21, miR-33, miR-34a, miR-146a, miR-155, and miR-223 and their related cardiovascular pathologies.

miRNAs have emerged as novel regulators of inflammatory processes with effects on the development of cardiovascular events (**Figure 1**). However, much work has to be performed to improve the use of miRNA mimics or inhibitors as new therapeutic tools or the use of miRNAs as prognostic or diagnostic biomarkers of adverse cardiovascular events in inflammatory diseases. This review only focused on the most relevant inflamma-miRs (**Table 1**), but studies have shown that many more miRNAs play roles in thromboinflammatory processes (**Table 2**).

**Table 1:** Role of selected inflamma-miRs in cardiovascular pathophysiology.

miRNA	Upstream regulation	Targets	Functional effect	Ref
miR-21	LPS	↓PDCD4	Activation of NF-κB or transcription of IL10	19
		↓ SORBS2	Worst outcome in septic cardiomyopathy	24
	Oscillatory shear stress	↓ PPARα	Endothelial cells inflammation	25
	Induced overexpression (miR-21 mimic)	↑ Akt/PKB	NO production, proliferation and apoptosis of endothelial cells	26
	Genetic ablation (miR-21^-/-^)	↓ ABCG1	Foam cell formation, larger atherosclerotic areas	28
miR-33	SREBP in situations of reduced lipid abundance	↓CPT1A ↓ HADHB ↓ CROT	Reduced cellular cholesterol metabolism	42
	Genetic ablation in macrophages (miR-33^-/-^)	─	Reduced lipid deposits, inflammation and plaque burden	44
	Genetic ablation in the liver (miR-33^-/-^)	─	Resistance to hepatic fibrosis and insulin	45
	─	↓ ABCA1	Increased cardiac fibroblast proliferation	47
			Regulated amyloid burden and inflammation in neurons and glia.	48
miR-34a	p53 (positive feedback loop)	↓ SIRT1	Increased acetylated p53 and modulation of apoptosis.	33
	Damaged cardiac microvascular endothelial cells by incubation with homocysteine	↓ NOTCH1	Increased apoptosis and decreased angiogenesis.	35
	Hypoxia in cardiomyocytes	↓ ZEB1	Increased apoptosis.	36
	Genetic ablation (miR-34a^-/-^) in macrophages	↑ ABCA1 ↑ ABCG1	Reduced cholesterol efflux, reverse cholesterol transport and inhibited atherosclerosis progression in an ApoE^-/-^ mouse model.	37
	Calcific aortic valve disease ↓ NOTCH1	↑ RUNX2	Increased calcium deposition of aortic valves and cardiac hypertrophy.	39
	Myocardial infarction and TGFβ1	↓ SMAD4	Progression of cardiac tissue fibrosis	40
miR-146a	NF-κB	↓ IRAK1 ↓ TRAF6	Fine-tune inflammatory cytokine production	53
	Genetic ablation (miR-146a^-/-^) and endotoxemic challenge	─	Exaggerated inflammation, multiorganic inflammation, myeloid proliferation, cancer and premature death.	54
	Induced overexpression (miR-146a mimic)	↓ NOX4	Preserved cardiac function and reduced the infarct zone and inflammatory response in an ischemic/reperfusion injury model.	55
	Ischemic/reperfusion injury (↓ miR-146a mitochondrial)	↑ Cyclophilin D	Mitochondrial dysfunction and cardiomyocyte apoptosis.	56
	Genetic ablation (miR-146a^-/-^) in fat-fed Ldlr^-/-^ mice	↑ SORT1	Increased proinflammatory cytokines and a reduction in atherosclerosis.	58
miR-155	Dietary compounds such as polyunsaturated fatty acids or arachidonic acid (↓ miR-155)	─	Anti-atherogenic effects.	66
	Inflammatory stimuli (NF-κB binds to the promoter of pri-miR-155)	↓ CARHSP1	Shift in macrophages to M1 phenotype.	
	LPS-induced sepsis Molecular clock	↓ BMAL1	Disturbed circadian function.	70
	TNF-α	↓ sGCβ1	Reduced contractile capacity of vascular smooth muscle cells and less vasorelaxation.	71
	ox-LDL in macrophages induced TRIF and ERK signaling.	↓ SOCS1	Regulation of macrophage inflammatory response.	72
	miR-155 antagomir (↓ miR-155)	↑ SOCS1		76
	Induced overexpression (miR-155 mimic)	↓ ABCA1 ↓ ABCG1	Reduced cholesterol efflux and increased foam cell formation.	64
	CTRP12 (↓ miR-155) ↑ ABCA1	↑ ABCG1	Inhibition of lipid accumulation and inflammatory response.	
	Acute myocardial infarction	─	Decreased apoptosis in cardiomyocytes.	76
miR-223	C/EBP-α or –β and NFI-A negative feedback loop	↓ NFI-A	Increased myeloid cell differentiation.	78
	Cell activation *via* TLRs	↓ NLRP3	Prevention of IL-1β production.	79
	Plasma HDL by SR-BI-induced lipid flux	Formation of HDL-miR-223 complexes	Control of cholesterol homeostasis and cell-cell intercommunication.	81
	─	↓ ICAM1	Regulates the NF-κB and MAPK signaling pathways in endothelial cells decreasing lipid deposition in the arterial wall and promoting endothelial activation.	84
	Genetic ablation (transplantation with bone marrow miR-223^-/-^ in mice)	↑ SP3 ↓ ABCA1 ↓ RETNLA	Increased atherosclerotic plaques size, lipid levels and IL-1β plasma levels.	85
	Atherosclerosis lesions	↓ MEK1	Stable atherosclerosis lesions and reduced levels of atherosclerotic markers.	86
	Pre-miR-223^-/-^ mice hearts	↑ IKKα pathway ↑ NLRP3	Higher inflammation and necroptosis.	87
	Induced by hypoxia	↓ KLF15	Increased oxidative stress, caspase-3 activation and ROS generation.	88
	─	P2Y_12_	Regulated platelet reactivity.	89
				90
			Biomarker of the response to antiplatelet drugs.	83, 92 93

↑, upregulated; ↓, downregulated; -, No target associated.

**Table 2:** miRNAs implicated in thromboinflammation.

Platelet function	miR-96 (119); miR-200b, miR-495, miR-107 (120); miR-376c (121); miR-223 (122); miR-126, miR-50, miR-191 (123); miR-10a/b (124); miR-326 (125); miR-331, miR-128, miR-500 (126).
NETosis	miR-146 (103, 111); miR-155 (104); miR-504 (127); miR-378a and miR-15b (128); miR-1696 (129); miR-16-5p (130).

One important point concerning the role of miRNAs in thromboinflammation, and other pathologies is their regulation and the factors that may modify their levels and/or function. In this review, we described how a variant affecting miR-146a, rs2431697, may impact the development of adverse cardiovascular events in several pathologies due to the downregulation of its expression (61, 111, 114). However, many variants affecting inflamma-miRs have been described (117) (131). Most of these miR-SNPs are associated with cancer, but it would be of interest to investigate whether these and other miR-SNPs can drive thromboinflammatory processes, as is the case for rs2431697.

Methylation is another process that may alter miRNA expression. Genes transcribing inflamma-miRs such as miR-21 and mir-145 have been shown to be hypermethylated in gonadotroph pituitary tumors (132). A recent review by Pajares et al. recapitulated how the methylation status of several inflamma-miRs may be potential biomarkers for diagnosis or prognosis in different types of cancer (133). Alternatively, mature inflamma-miRs such as miR-17-5p, miR-21-5p, and miR-let-7a-5p may be methylated in gastrointestinal cancers, and these posttranscriptional modifications may impact their stability and the regulation of some of their targets (134). Thus, future studies exploring whether miRNA genes or mature miRNA methylation status occurs in inflammatory diseases that lead to thromboinflammatory processes may be of interest.

A few years ago, adenosine-to-inosine (A-to-I) editing, which consists of the conversion of adenosine to inosine in double-stranded RNA by adenosine deaminases acting on RNA (ADAR) 1 and 2, was described in miRNA precursors, which led to reduced expression or altered function of the mature forms of miRNAs (135). Inflamma-miRs such as miR-33 and miR-211/222 have been shown to be affected by A-to-I editing. Thus, miR-33 biogenesis in the brain may be controlled by this editing mechanism, since A-to-I editing of pri-miR-33 inhibits cleavage by Drosha (136). On the other hand, ADAR2-mediated editing of mir-222 and mir-221 precursors has been shown to inhibit their maturation in glioblastoma (137). In the cardiovascular field, van der Kwast et al. showed that the A-to-I editing of pri-miR-487b modified the targetome of the new mature edited miR-487b, which was then enriched in multiple proangiogenic pathways (138). This edited form of miR-487b was increased in murine muscle tissue during postischemic neovascularization (138). Although miR-487b is not an inflamma-miR and its function is related to angiogenesis, this study encourages the search for inflamma-miRs that may be edited and be involved in thromboinflammatory processes in cardiovascular pathologies.

There are factors that may dysregulate the expression of mature miRNAs, which may be critical in the development of adverse cardiovascular events due to thromboinflammatory processes. Efforts should be made to find the correct models and adequate patient cohorts to further demonstrate the implications and functions of miRNAs in cardiovascular pathologies associated with inflammatory diseases.

## Author contributions

LZ-M and SÁ wrote the original draft. AR-G, SC-T, ML reviewed the manuscript. RG-C and CM edited and supervised the manuscript. All authors contributed to the article and approved the submitted version.
